# ColoFinder: a prognostic 9-gene signature improves prognosis for 871 stage II and III colorectal cancer patients

**DOI:** 10.7717/peerj.1804

**Published:** 2016-03-14

**Authors:** Mingguang Shi, Jianmin He

**Affiliations:** 1 School of Electric Engineering and Automation, Hefei University of Technology, Hefei, Anhui, China; 2 School of Management, Hefei University of Technology, Hefei, Anhui, China

**Keywords:** Colorectal cancer, The 9-gene signature, Random survival forest

## Abstract

Colorectal cancer (CRC) is a heterogeneous disease with a high mortality rate and is still lacking an effective treatment. Our goal is to develop a robust prognosis model for predicting the prognosis in CRC patients. In this study, 871 stage II and III CRC samples were collected from six gene expression profilings. ColoFinder was developed using a 9-gene signature based Random Survival Forest (RSF) prognosis model. The 9-gene signature recurrence score was derived with a 5-fold cross validation to test the association with relapse-free survival, and the value of AUC was gained with 0.87 in GSE39582(95% CI [0.83–0.91]). The low-risk group had a significantly better relapse-free survival (HR, 14.8; 95% CI [8.17–26.8]; *P* < 0.001) than the high-risk group. We also found that the 9-gene signature recurrence score contributed more information about recurrence than standard clinical and pathological variables in univariate and multivariate Cox analyses when applied to GSE17536(*p* = 0.03 and *p* = 0.01 respectively). Furthermore, ColoFinder improved the predictive ability and better stratified the risk subgroups when applied to CRC gene expression datasets GSE14333, GSE17537, GSE12945and GSE24551. In summary, ColoFinder significantly improves the risk assessment in stage II and III CRC patients. The 9-gene prognostic classifier informs patient prognosis and treatment response.

## Introduction

According to the American Joint Committee on Cancer (AJCC) stage of colorectal cancer (CRC), 5-year survival rates are 82.5% for stage II and 59.5% for stage III CRC patients respectively (O’Connell, Maggard & Ko, 2004). Approximately, 5-year stage-specific survivals are 72.2% for stage IIB (T-stage 4, lymph node–negative) and 83.4% for stage IIIA (T-stage 1–2, lymph node–positive) CRC patients. Stage IIB is significantly poorer survival than stage IIIA (O’Connell, Maggard & Ko, 2004). In clinical trials, there are approximately 20% of stage II CRC patients who do not make the expected benefits from the adjuvant chemotherapy (CTX). On the other hand, 42–44% of stage III patients treated by surgery alone do not recur in 5 years (Ragnhammar et al., 2001). Based on these observations, it underlines the need for accurate assessment of recurrent risk for stage II and III CRC patients in order that high-risk patients could be treated with adjuvant CTX, but low-risk patients avoid unnecessary adjuvant CTX.

Several protein and genomic biomarkers have been used as prognostic and predictive markers to refine the prognostic information of CRC. Microarray-based gene expression profiling has showed great potential in identifying sub-network biomarkers (Shi, Beauchamp & Zhang, 2012; Shi et al., 2014), molecularly distinct subtypes (Oh et al., 2012), transcriptional subtypes (Zhu et al., 2013), 34-gene expression signature (Smith et al., 2010), 18-gene expression signature (Salazar et al., 2011), 13-gene expression signature (Ag˚esen et al., 2012), 7-gene expression signature (Sveen et al., 2012) and 4-gene expression signature (Zou et al., 2015) for survival analysis in CRC patients. In-depth proteomics to stool samples from CRC patients and healthy controls was applied to identify tumor-specific protein based biomarkers for the early detection of CRC (Bosch et al., 2012). Plasma MicroRNAs are very potential as novel noninvasive biomarkers for early detection of CRC (Huang et al., 2010). Of all these markers, KRAS was the first biomarker integrated into clinical practice for CRC (Van Schaeybroeck et al., 2011). The prognostic/ predictive markers such as BRAF, PIK3CA, PTEN, CEA and CA199 were potential for the implementation of these biomarkers into routine clinical trials.

Many previous analysis for CRC gene expression signatures were often limited to small sample sizes and the lack of independent sample test (Lu et al., 2009). To overcome the shortcomings, larger sample studies were applied to verify the predictive value of prognostic gene expression signatures. Nevertheless, the main limitations of the proposed assays were insufficient prognostic messages for signatures, which possibly resulted in large quantities of signatures and the weak robustness. Overall, the clinical application of these tests were restrained from inadequate independent validation (Sveen et al., 2013).

In this study, we selected 9-gene expression signatures as prognostic and predictive DNA markers to develop the prognosis model. Our hypothesis was that the functionally important mutated genes with CRC recurrence improved the cancer prognosis and clinical outcome. We used a 9-gene signature-based Random Survival Forest (RSF) prognosis model to develop ColoFinder. The prognosis model was then externally validated in five independent gene expression datasets to prove its effectiveness. The results demonstrated that ColoFinder improved the predicted performance of prognosis and provided the concise testing result for general application in clinical trials.

## Materials and Methods

### Study patients

We derived the expression profiles from 871 colorectal cancer patients with stage II and stage III samples to test associations between the 9-gene signature and clinical outcomes. Raw microarray data were obtained from six publicly available CRC gene expression datasets with available clinical information including NCBI Gene Expression Omnibus (GEO) GSE39582 (Marisa et al., 2013) (461 samples with relapse-free survival); GSE17536 (Freeman et al., 2012; Smith et al., 2010) (111 samples with relapse-free survival); GSE14333 (Jorissen et al., 2009) (67 samples with relapse-free survival); GSE17537 (Freeman et al., 2012; Smith et al., 2010) (34 samples with relapse-free survival); GSE12945 (Staub et al., 2009) (38 samples with relapse-free survival) and GSE24551 (Sveen et al., 2011) (160 samples with relapse-free survival). We used the largest CRC gene expression dataset GSE39582 (*n* = 461) as training dataset and the other five gene expression datasets as validation dataset. Specifically, we combined GSE17537 with GSE12945 as an independent validation dataset for study. The sampling distributions with clinical and demographic information of CRC samples were listed in Table 1.

**Table 1:** The microarray gene expression datasets used in the study (N=871).

	All trails (*N* = 871)	GSE39582 (*N* = 461)	GSE17536 (*N* = 111)	GSE14333 (*N* = 67)	GSE17537 (*N* = 34)	GSE12945 (*N* = 38)	GSE24551 (*N* = 160)
Stage							
II	479	260	55	37	15	22	90
III	392	201	56	30	19	16	70
Recurrence							
No	620	322	80	54	31	35	98
Yes	251	139	31	13	3	3	62
Age (years)							
Range	22–94	22–97	26–92	30–92	47–94	NA	NA
Median	NA	69	67	70	63	NA	NA
Gender							
Male	371	258	53	41	19	NA	NA
Female	302	203	58	26	15	NA	NA
Adjuvant CTX							
0	302	258 (203 II + 55 III)	NA	44(33 II + 11 III)	NA	NA	NA
1	225	202(56 II + 146 III)	NA	23(4 II + 19 III)	NA	NA	NA
#genes	NA	19825	19468	19468	19468	12694	16733

The Robust MultiChip Analysis (RMA) algorithm (Irizarry et al., 2003) was employed with quantile normalization and log2-transformation for normalizing and summarizing probe-level intensity. The probe set identifiers (IDs) were mapped to gene symbols based on the mapping file from GEO database and the gene with the largest interquartile range (IQR) was used for study when multiple probe sets were mapped to the same gene. For making comparable gene expression level, the *z*-score transformation was used to standardize the expression values of each gene.

### The prognostic gene expression signatures

We hypothesized that the functionally important mutated genes improved the predicted performance of cancer prognosis and clinical outcome. We selected the CRC recurrence related mutated genes with functional importance, developed the prognosis model and improved the prognosis of CRC. The selected gene expression signatures comprised a small set of the 9-gene signature including APC, MLH1, MSH2, MSH6, TP53, TGFBR2, SMAD4, KRAS and PTEN gene. The 9-gene signature was composed of oncogenes and tumor-suppressor genes which are associated with CRC (Markowitz & Bertagnolli, 2009). Specifically, the 9-gene signature was composed of prognostic and predictive DNA markers in CRC (Markowitz & Bertagnolli, 2009). APC degraded *β*-catenin and inhibited its nuclear localization which activated the Wnt signaling pathway. The germ-line defects in mismatch-repair genes such as MLH1, MSH2, and MSH6 were responsible for the colorectal cancer. The mutation of TP53 inactivated the p53 pathway which is the key genetic step in colorectal cancer. Somatic mutations inactivated TGFBR2 for colorectal cancer, and the mutational inactivation of TGF-*β* signaling was the key step for the progression of colorectal cancer. SMAD4, along with proteins SMAD2 and SMAD3, were critical for transforming growth factor *β* pathway signaling. KRAS activated the mitogen-activated protein kinase (MAPK) signaling pathway for colorectal cancers. PTEN acted as a tumor suppressor gene through the action of its phosphatase protein product and promoted the activation of PI3K pathway signaling, which leaded to cell-survival signaling and apoptosis suppression.

### Random survival forest (RSF) prognosis model

The R-package randomSurvivalForest was used to develop the random survival forest (RSF) prognosis model (Ishwaran et al., 2008). The 9-gene signature was applied for training set to develop the prognosis model. The model had two parameters *ntree* and *mtry*, where *ntree* was the number of trees in the forest and *mtry* was the number of variables randomly selected for splitting at each node. For the tuned parameters, we let *ntree* vary among the candidate set {10, 20, 30, 40, 50, 60, 70, 80, 90, 100} and *mtry* vary among the candidate set {1,3,5,7,9} to form different parameter combinations. Each combination of parameter choices was assessed using 5-fold cross-validation, and the parameters with best cross-validation AUC (the Area under the Receiver Operating Characteristic Curve) were discovered. The final RSF model was then trained on the whole training set using the optimal parameters, and tested on the independent validation dataset for the evaluation of AUC.

### The validation of independent gene expression data sets

The ColoFinder was then tested in the independent cohorts GSE17536, GSE14333, GSE17537, GSE12945 and GSE24551. The ensemble mortality for individual samples, which we called 9-gene signature recurrence scores, were calculated with test cohorts. A higher score indicated higher risk with shorter survival time. In the test dataset, the median score was used to stratify patients into high-risk and low-risk score groups in all cohorts.

### Statistical analysis

We developed ColoFinder to derive AUC and standard Kaplan–Meier survival curves from training dataset (Fig. 2). The prognostic 9-gene signature was validated in the test dataset for deriving the recurrence scores of each sample, and the performance was then assessed with AUC and hazard ratios (HRs). We compared the 9-gene signature with the published gene expression signatures and the results demonstrated that the 9-gene signature consistently outperformed the available gene expression signatures (Figs. 3 and 4). Cox regression analysis was performed to assess the prognostic value of the 9-gene signature with relapse-free survival probability (Table 2). The Cox regression models were built and HR was calculated with R survival package. All statistical tests were two-sided and *p* value less than 0.05 was considered statistically significant.

## Results

### Overview of the ColoFinder development and evaluation workflow

**Figure 1:**
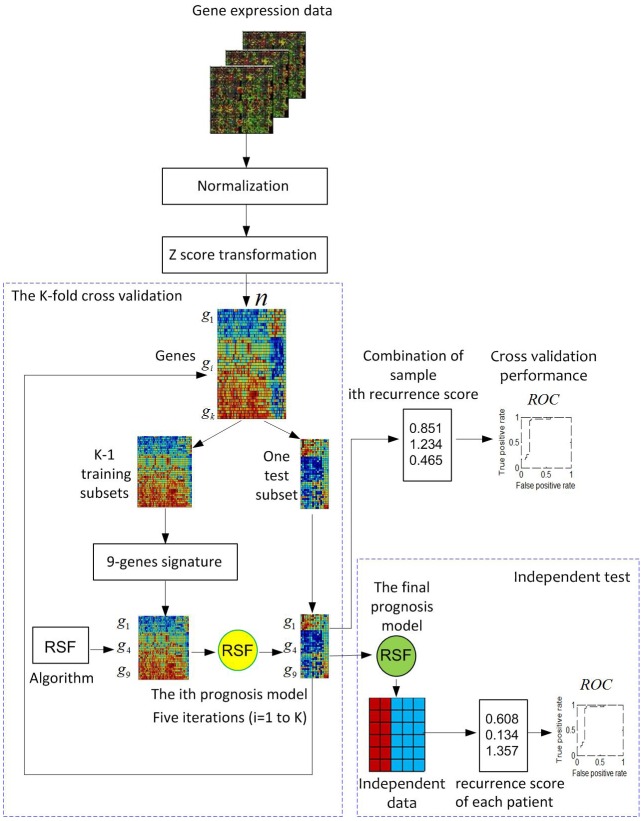
Pipeline for the development and evaluation of the ColoFinder. Gene expression data are chosen, normalized, and *z*-score transformed. A *K*-fold cross-validation is used for the development of the random survival forest (RSF) prognosis model (*K* = 5). The 9-genes signature was selected to develop the RSF prognosis model. A fully-developed model based on the optimal parameters identified in cross-validation was then evaluated in an independent dataset.

Figure 1 illustrates the overview of the ColoFinder development and evaluation workflow. Microarray gene expression data of CRC are collected, normalized, and *z*-score transformed. A K-fold cross-validation is used for RSF model development with the samples randomly partitioned into K subsets. A single subset is retained as a temporary test subset and the remaining K-1 subsets are used as a temporary training set. Data on the 9-gene signature for samples in the training set is used to parameterize the RSF model algorithm. The parameterized model is then used to predict samples in the test subset. The cross-validation process is then repeated K times, with each of the K subsets used exactly once as the test subset. The K results from the subsets then can be combined to produce a single estimation. Fully developed RSF model based on the optimal parameters identified by in the cross-validation process is then validated by an independent dataset. The performance was evaluated based on AUC.

**Figure 2:**
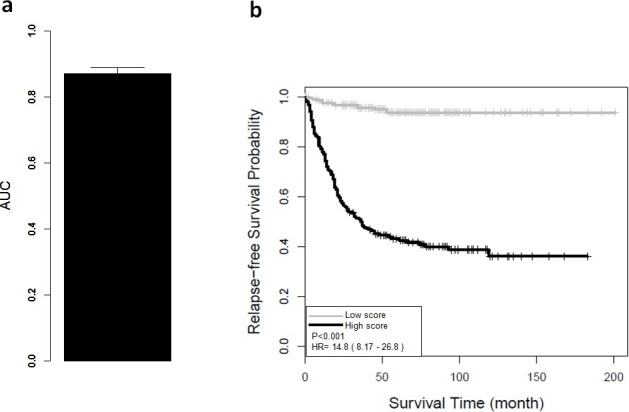
Performance of ColoFinder with 5-fold cross-validation for training data set. (A) Receiver operating characteristic analysis showed significant ability to discriminate between high-risk and low-risk groups in the CRC GSE39582 cohort. The average AUC is 0.87 (95% CI [0.83–0.91]) with 5-fold cross validation. (B) Kaplan–Meier survival curves for patient subgroups identified in GSE39582. It showed a significant difference in distant relapse-free survival for high-risk and low-risk groups of CRC patients.

**Figure 3:**
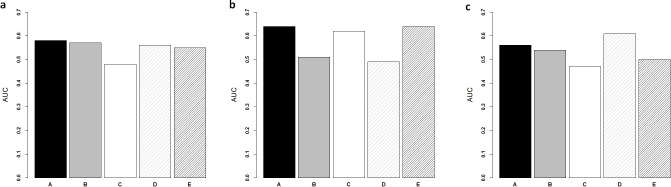
Comparison of ColoFinder and N-genes based RSF model applied on the CRC patients. Receiver operating characteristic analysis of the predictions for three independent test cohorts. (A) GSE14333. (B) B, GSE17537+ GSE12945. (C) GSE24551. (A) 9-genes signature, (B) 18-genes, (C) 34-genes, (D) 13-genes, (E) 6-genes.

**Figure 4:**
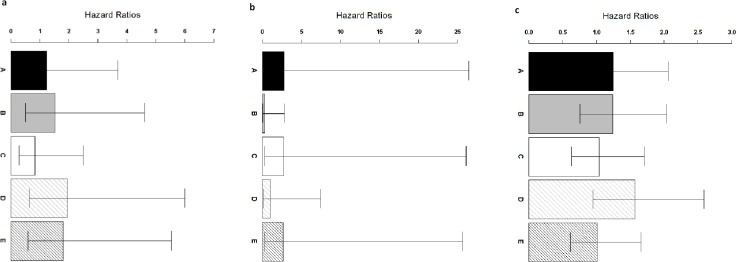
Comparison of ColoFinder and N-genes based RSF model applied on the CRC patients. Hazard ratios of the predictions for three independent test cohorts with 95% confidence intervals. (A) GSE14333. (B) B: GSE17537+ GSE12945. (C) GSE24551. A: 9-genes signature, B: 18-genes, C: 34-genes, D: 13-genes, E: 6-genes.

**Table 2:** Univariate and multivariate Cox proportional hazard regression analyses of relapse-free survival in GSE17536.

	Univariate		Multivariate	
	*p* value	HR(95% CI)	*p* value	HR(95% CI)
Age	0.25	0.98 (0.96–1.01)	0.14	0.98 (0.95–1.01)
Gender (M or F)	0.72	0.88 (0.43–1.78)	0.88	1.06 (0.49–2.30)
AJCC STAGE (II, III)	0.07	1.96 (0.94–4.09)	0.08	1.97 (0.93–4.16)
ColoFinder	0.03	0.44 (0.21–0.93)	0.01	0.34 (0.15–0.77)

**Notes.**

AJCC American Joint Committee on Cancer F female M male

### Performance of ColoFinder for training dataset

The AUC and Kaplan–Meier survival curves were derived from ColoFinder. In this study, the best performed parameters were used to develop the RSF prognosis model where *ntree* = 50 and *mtry* = 3 respectively 9-gene signature recurrence score based prognosis model was calculated for individual samples of the training data set GSE39582. Figure 2A depicted the average AUC of RSF model from the 5-fold cross-validation studies. The receiver operating characteristic analysis showed good sensitivity and specificity with average AUC of 0.87 (95% CI [0.83–0.91]). Based on the recurrence score, the patients were divided into two groups, including a low-risk group with below-median scores and a high-risk group with above-median scores. As shown in Fig. 2B, the low-risk group had significantly better relapse-free survival (hazard ratio (HR), 14.8; 95% CI [8.17–26.8]; *P* < 0.001) than the high-risk group. The relapse-free survival at 3 years was 59% for the high-risk group compared with 100% for the low-risk group.

### ColoFinder significantly improved association with relapse-free survival

The univariate and multivariate Cox proportional hazards regression analyses were applied to independent test cohort GSE17536 for evaluating the prognostic value of 9-gene signature recurrence score. ColoFinder provided the recurrence score from 48 to 351 for each sample in test cohort. With multivariate logistic regression including patient age at diagnosis, gender, AJCC stage and the 9-gene signature recurrence score, we found that in the GSE17536 test cohort the 9-gene signature recurrence score was significantly associated with relapse-free survival (*p* = 0.01) (Table 2). In univariate Cox analyses, the 9-gene signature recurrence score still maintained the significance associated with relapse-free survival (*p* = 0.03) (Table 2). Thus, the 9-gene signature recurrence score was more statistically significantly associated with relapse-free survival than standard clinical and pathologic covariates.

### ColoFinder better stratified three independent series of CRC patients

ColoFinder was tested on the independent validation dataset, and then evaluated with the AUC and hazard ratios. The 9-gene signature recurrence score was derived for each patient in the three independent validation cohorts respectively. Figure 3 illustrated the performance of AUC on independent validation dataset. As shown in Fig. 4, the estimated hazard ratio (HR) with 95% confidence intervals was calculated for the validation data sets. The value of HR was used to evaluate the clinically significant difference between high-risk and low-risk groups. The bigger the value of HR is, the better the statistical significance is.

The 9-gene signature was validated on the test cohort GSE14333, resulting in an ROC curve with AUC of 0.58 (Fig. 3A). A total of 25 of 33 patients did not develop distant relapse in the predicted high-risk group, while 5 of 34 patients developed distant relapse in the predicted low-risk group. The 9-gene signature recurrence score ranged from 38.8 to 413 among all patients in the test cohort GSE14333, and the 9-gene signature was associated with distant relapse-free survival (HR, 1.24 95% CI [0.42–3.69]) (Fig. 4A). Patients in the low-risk group had a median relapse-free survival time of 41 months compared with 35 months in the high-risk group.

Investigation of the 9-gene signature recurrence score in patients from two additional cohorts confirmed its association with distant relapse-free survival. Firstly, for the independent GSE17537 and GSE12945 test cohorts, the 9-gene signature recurrence score was also associated with relapse-free survival in patients (HR, 2.75 95% CI [0.287–26.5]) (Fig. 4B). A total of 33 of 36 patients did not develop distant relapse in the predicted high-risk group, while one of 36 patients developed distant relapse in the predicted low-risk group. The median relapse-free survival time was 46 months in the cases with low-risk group compared with only 44 months in the cases with high-risk group. Furthermore, the 9-gene signature was validated on the test cohorts, resulted in an ROC curve with AUC of 0.64 (Fig. 3B). Secondly, starting from independent test cohort data set GSE24551, the 9-gene signature recurrence score was consistently associated with relapse-free survival in patients (HR, 1.25 95% CI [0.758–2.06]) (Fig. 4C). Specifically, the 9-gene signature was validated on the test cohort GSE24551, which led to the ROC curve with AUC of 0.56 (Fig. 3C). In summary, ColoFinder improved the predictive ability for independent test cohorts and better stratified the risk subgroups of CRC.

### The 9-gene signature compared with the published gene expression signatures

To further evaluate the significance of the 9-gene signature, the prognostic potential from the 9 gene signatures was compared with that from existing prognostic gene signatures ( Ågesen et al., 2012; Salazar et al., 2011; Smith et al., 2010; Zou et al., 2015). The N-genes (18-genes Salazar et al., 2011, 34-genes Smith et al., 2010, 13-genes  Ågesen et al., 2012 and 6-genes (CRP, IL10, IL2, IL8, LPA, TNF) Zou et al., 2015) signature based RSF model were trained respectively on the gene expression dataset GSE39582 and then tested on the independent test datasets. Figure 3 depicted the AUC when the N-genes signature score was derived for each patient in the three independent test cohorts. For GSE14333, the 9-gene signature achieved 1.8%, 20.8%, 17.9% and 5.5% increase respectively in AUC as compared to 18-genes, 34-genes, 13-genes and 6-genes signatures respectively (Fig. 3A). For GSE17537 and GSE12945, 25.5%, 3.2% and 30.6% increase in AUC were achieved with the 9-gene signature in comparison with 18-genes, 34-genes and 13-genes (Fig. 3B). For GSE24551, the 9-gene signature achieved 3.7%, 19.1% and 12% increase respectively in AUC as compared to 18-genes, 34-genes and 6-genes signatures respectively (Fig. 3C). Specifically, the 13-genes signature achieved 8.9% increase in comparison with the 9-gene signature (Fig. 3C).

The 9-gene signature achieved an HR of 2.75 95% CI [0.287–26.5] in the GSE17537 and GSE12945 datasets, which was 841%, 1.4%, 162% and 3% higher than that from 18-genes, 34-genes, 13-genes and 6-genes signatures respectively (Fig. 4B). A increase of 0.8%, 20.2% and 23.7% in the GSE24551 dataset was achieved with the 9-gene signature in comparison with 18-genes, 34-genes and 6-genes (Fig. 4C). Specifically, the 13-genes signature achieved 25.6% increase in comparison with 9-gene signature (Fig. 4C). Interestingly, The 13-genes signature achieved an HR of 1.94 95% CI [0.628–6] in the GSE14333 dataset, which was 56.5%, 27.6%, 134.5% and 7.7% higher than that from 9-gene, 18-genes, 34-genes and 6-genes signatures, respectively (Fig. 4A).

The Cox proportional hazards regression analyses were applied to GSE17536 for evaluating the prognostic value of N-genes signature score. With multivariate logistic regression including patient age, gender, AJCC stage and the N-genes signature score, we found the predicted outcome with N-genes signature score in the test cohort (18-genes, *p* = 0.34; 34-genes, *p* = 0.06; and 13-genes, *p* = 0.07). In univariate Cox analyses, the N-genes signature score was used to predict the association with relapse-free survival respectively (18-genes, *p* = 0.46; 34-genes, *p* = 0.09; and 13-genes, *p* = 0.04). Thus, the 9-gene signature consistently outperformed the available gene expression signatures.

## Discussion

A major conclusion from this study was that ColoFinder was able to predict the prognosis for stage II and III CRC patients. ColoFinder provided the accurate prognostic model for predicting the performance of external validation cohorts from different countries, races and microarray platforms. The results demonstrated that our model provided useful predictive information regarding the prognosis for CRC patient subgroups. Our analysis used the large gene expression datasets with 871 CRC samples. The validated 9-gene signature provided extra value compared with standard clinical and pathologic covariates. To test the generality of the 9-gene signature, we applied the Recursive Partitioning and Regression Trees (RPART) model to test the association with CRC prognosis. Performance results are available in the Supplemental Information 1.

A data-driven strategy has been popularly made for gene signature search strategy in analyzing gene expression dataset. To prioritize the gene signature of gene expression data, several search strategies have been provided with unsupervised hierarchical clustering analysis (Oh et al., 2012), the nearest mean classifier (Salazar et al., 2011) and Cox proportional hazards survival modeling based on lasso estimation ( Ågesen et al., 2012). In this study, we selected the gene signature which was critical for promoting CRC recurrence to construct the prognosis model. We aimed to analyze the genomic alterations with impact on prognosis and survival in CRC. The implementation process of genes relevant for CRC was knowledge driven, consisting of mismatch repair proteins, proteins of the EGFR-KRAS-PTEN cascade, APC and b-catenin of the WNT pathway. The results demonstrated that ColoFinder had the potential to predict the prognosis of CRC patients.

Random Forest (RF) was a non-parametric ensemble tree learning method which had been generally used for gene expression data analysis (Breiman, 2001; Díaz-Uriarte & De Andres, 2006; Statnikov, Wang & Aliferis, 2008). Random Survival Forest (RSF), the extension of RF method, was ensemble tree method for analyzing the right-censored survival data (Ishwaran et al., 2008). The advantage of this method was to model non-linear effects and multiple interactions among complex features. Although RSF had been successfully utilized for cancer pathway hunting and genomic analysis (Chen & Ishwaran, 2013; Ishwaran et al., 2011; Ishwaran et al., 2014), the overfitting of this high-dimensional survival analysis model reduced the significance of the genomic predictor when applied to an independent dataset ( Ågesen et al., 2012). In this study, we developed the RSF prognosis model with small set of gene signatures to effectively restrain this overfitting of survival model.

Several tests have now been clinically provided for CRC survival analysis, such as ColoPrint (Salazar et al., 2011), Oncotype DX (O’Connell et al., 2010), ColoGuideEx ( Ågesen et al., 2012) and ColoGuidePro (Sveen et al., 2012). The proposed prognostic gene signatures with small set predicted CRC recurrence and provided useful insights into patient response from adjuvant CTX. All these tests have demonstrated that the gene signatures have prognostic value in independent patient series across different microarray platforms. Furthermore, the test Oncotype DX has been clinically validated as a prognostic signature for stage II CRC patients in a subsequent large clinical study (NSABP C-07) (Yothers et al., 2013). In addition, the microsatellite instability (MSI) status of the tumor has been used for stage II CRC treatment. The patients with high level of MSI have a favorable prognosis and improved treatment effect (Boland et al., 1998; Gryfe et al., 2000). The mutations activating the RAS/RAF signaling pathway were also predictive and prognostic indicators in CRC patients (Benvenuti et al., 2007).

The univariate analysis was performed using the Cox proportional hazards regression model in independent test cohort GSE17536 to evaluate the single effects of each marker. The single variable associated with relapse-free survival was APC (*p* = 0.27), MLH1 (*p* = 0.07), MSH2 (*p* = 0.91), MSH6 (*p* = 0.62), TP53 (*p* = 0.46), TGFBR2 (*p* = 0.44), SMAD4 (*p* = 0.27), KRAS (*p* = 0.07) and PTEN (*p* = 0.35) respectively. As shown in Table 2, the 9-gene signature recurrence score maintained the significance associated with relapse-free survival in univariate Cox analyses (*p* = 0.03).

This study has several limitations. The RSF prognosis model has some drawbacks when determining the variable importance. For data including categorical variables with different number of levels, RSF is biased in favor of those attributes with more levels. Although ColoFinder achieved better performance than N-genes based RSF model when applied to independent datasets, the AUC is slightly poor and smaller than 0.65. We also noticed that the AUC of the 9-gene signature in the training set was 0.87, but the values of AUC dropped to smaller than 0.65 in the validation sets. Similarly, the hazard ration (HR) dropped from 14.8 in the training set to smaller than 3.0 in the validation sets. A possible explanation is the different class proportions between the training and the validation datasets, given the small sample size in both datasets. For example, the proportion between recurrence and non-recurrence is 1:2.3 in GSE39582, 1:4.2 in GSE14333, 1:11 in GSE17537 and GSE12945 and 1:1.6 in GSE24551, respectively. Although the class proportion can be well maintained in a cross-validation, it is not controlled for independent validation sets.

In conclusion, we developed ColoFinder to predict the prognosis of cancer patients and the prognosis model improved the prognosis for stage II and III CRC patients. Strong risk stratification was realized in five independent series of cancer patients from different microarray platforms. The results demonstrated that the 9-gene signature could improve the risk assessment and aid in clinical practice for CRC patients.

## Supplemental Information

10.7717/peerj.1804/supp-1 Supplemental Information 1 ColoFinder: a prognostic 9-genes signature improve prognosis for 871 stage II and III colorectal cancer patients Supplementary file.

## Additional Information and Declarations

### Data Availability

The following information was supplied regarding data availability: Raw data are publicly available in the Gene Expression Omnibus(GEO) datasets (accession number GSE39582, GSE17536, GSE14333, GSE17537, GSE12945 and GSE24551).
