# Supplementary material for: ColoFinder: a prognostic 9-gene signature improves prognosis for 871 stage II and III colorectal cancer patients
Source: PeerJ. 2016 Mar 14;4:e1804. doi: 10.7717/peerj.1804 (PMC4793313; doi:10.7717/peerj.1804)
Supplement: Supplemental Information 1 — Supplementary file. [file peerj-04-1804-s001.docx]

**ColoFinder: a prognostic 9-genes signature improve prognosis for 871 stage II and III colorectal cancer patients**

**Mingguang Shi, Jianmin He**

**Analysis of RPART model**

An R implementation of the Recursive Partitioning and Regression Trees (RPART) available in the RPART package was employed for RPART model development([Therneau et al. 2010](#_ENREF_1)). Standard classification and regression tree analysis was used to build the RPART prognostic model based on 9-genes signature in the GSE39582 cohort. We first evaluated the performance of the RPART model using the leave-one-out cross-validation (LOOCV) method. The model was then evaluated in an independent data set. AUC and HRs were also used to evaluate the predicted performance.

**Analysis of 9-genes RPART model for CRC cohort**

The RPART prognosis model based on 9-genes signature was build in the GSE39582 cohort with LOOCV method. The receiver operating characteristic (ROC) curve showed good sensitivity and specificity with AUC of 0.86 (Figure S1a). As shown in Figure S1b, the high-risk group had significantly worse relapse-free survival (hazard ratio [HR], 9.72; 95% confidence interval [CI], 6.1 - 15.5; *p*<0.001) than the low-risk group. The relapse-free survival at 3 years was 50% for the high-risk group compared with 92% for the low-risk group.

**Figure S1 Performance of RPART prognosis model for training data set GSE39582.**  (a) Receiver operating characteristic analysis with the 9-genes RPART model showed significant ability to discriminate between high-risk and low-risk groups in the GSE39582 cohort (AUC=0·86). (b) Kaplan-Meier survival curves for patient subgroups identified in GSE39582 using 9-genes RPART model. It showed significant difference in distant relapse-free survival for high-risk and low-risk groups of CRC patients.

**RPART prognosis model applied to three independent series of CRC patients**

The 9-genes signature was validated on the test cohort GSE14333, resulting in an ROC curve with AUC of 0.52 (Figure S2a). The 9-genes signature scores were associated with distant relapse-free survival (HR, 1.31 [95% CI, 0.433 - 3.94]) (Figure S2b). The 9-genes signature was validated on the test cohorts GSE17537 and GSE12945, resulting in an ROC curve with AUC of 0.54 (Figure S2a). For the independent GSE17537 and GSE12945 test cohorts, 9-genes signature scores were also associated with relapse-free survival in patients (HR, 0.96 [95% CI, 0.135 - 6.83]) (Figure S2b). The 9-genes signature was validated on the test cohort GSE24551, resulting in an ROC curve with AUC of 0.58 (Figure S2a).Starting from independent test cohort data set GSE24551, 9-genes signature scores were consistently associated with relapse-free survival in patients (HR, 1.35 [95% CI, 0.818 - 2.23]) (Figure S2b).

**Figure S2 Predictive performance of the RPART prognosis model on the three independent test cohorts.** (a) Receiver operating characteristic analysis of the predictions for three independent series stratified by the 9-genes RPART model. A: GSE14333, B: GSE17537+ GSE12945, C: GSE24551. (b) Hazard ratios of the 9-genes RPART model on each validation data sets with 95% confidence intervals. A: GSE14333, B: GSE17537+ GSE12945, C: GSE24551.

1. Therneau TM, Atkinson B, Ripley MB: **The rpart package**. In*.*; 2010.
